# 

**Published:** 1846-01

**Authors:** 


### INDEX.

Abscess, cases of, connected with the urinary bladder, 123
Abscess of the left lung, thickening and partial ulceration of the pericardium, &c., by A. B. Smith, M.D. 197
Abscess, pelvic, 250
" fistulous, case of, in the liver, 514
" pelvic, case of, opened through the rectum, 506
Abscesses, on the formation of, 690
Abdominal tumour, an account of an enormous, by Dr. A. B. Longshore, 217
Absorption of chyle, experiments and observations on the, 324
Abortion, trial for the attempt to procure, 443
Acid irritation of the kidneys, a curious effect of, 378
Acute glossitis, a case of, 450
Aconitum napellus, an inquiry into the physiological and medicinal properties of the, by Dr. A. Fleming, 542
Adams's seven books of Paulus Ægineta, 561
Adulterations of various substances used in medicine and the arts, with the means of detecting them; intended as a manual for the physician, the apothecary, and the artisan, by L. C. Beck, M. D., 715
Address to the medical profession, in relation to the objects of the national medical association, by the committee appointed for that purpose, 748
African fever, 69
Alkaline carbonates, on the question whether they exist in the blood, 212
Allinson's case of ovarian dropsy, 336
Alienation, mental, case of, 565
Albumen, on a newly discovered characteristic of, 369
Alterations of names, 417
Albuminous nephrites in pregnant women, 631
Amputation in private practice, cases of, by T. F. Betton, M. D. 93
Ammonia, phosphate of, by C. Voigt, M. D. 289
Ammoniacal blistering ointment, Dr. Goudret's, 377
Animal chemistry, with reference to the Physiology and Pathology of man, 27
Anatomy of the human body, a pocket atlas descriptive of the, 44
Anatomy and physiology, Ranking's report on the progress of, 48, 111
Anatomy, pathological, 200
Anatomy and histology, special, 657
Anatomy and physiology, &c., a manual of examinations upon, by J. L. Ludlow, A. M., M. D., 658
Anatomy, a system of, for the use of students of medicine, by C. Wistar, M. D., 650
Announcement, annual, and catalogues, 110
Announcement, annual, of the Medical Department of the University of New York, 662
Announcement, annual, of the Medical Department of Pennsylvania College, 664
Annual Reports, 176
Annalist, the, a new medical journal in New York, 660
Aneurism, 629
" two cases of, 135
" popliteal, two cases of, 523
Antagonism of ague and of pulmonary consumption, M. Bricheteau on the, 248
Animal chemistry, with reference to the physiology and pathology of man, 351
Antidotes to poisons, abstract of a lecture on, 370
Anus, on the submucous division of the sphincter in affections of the, 506
Antimony, experiments on, 702
Arthur's popular treatise on the teeth, 108, 416
Artificial warts, 270
Arsenic, poisoning by, 688, 690
Asiatic cholera, 379, 419
Astragalus, dislocation of the, and reduction, 632
Auscultation, practice of, a clinical introduction to the, 298
Auscultation, mediate, a treatise on, and on diseases of the lungs and heart, by R. T. H. Laënnec, 343
Baker's cases illustrative of the ill effects of Onanism, 451
Barlow, on man's power over himself to prevent or control insanity, 596

Bark and leaves of the olive-tree, on the use of the, 742
Betton's cases of amputation in private practice, 93
Beck, on adulterations of various substances used in medicine and the arts, with the means of detecting them; intended as a manual for the physician, the apothecary, and the artisan, 715
Bellingham's observations on the employment of compression in aneurism, 721
Bell, on the use of sulphate of quinine in typhoid fever, 275, 402
Belladonna, atropa, or deadly nightshade, poisoning with the berries of, 745
Bibliographical notices, 27, 46, 160, 221, 289, 343, 407, 466, 542, 587, 647, 706
Bilious Remitting fever, on the treatment of, by A. Livezey, A. M., M. D., 94
Bile, yellow coloring matter of the, 202
Binoxide of protein, on the supposed, 245
Billet doux to Baron Liebig, 582
Bloomingdale Asylum for the insane, twenty-fifth annual report of, 225
Blistering ointment, Dr. Goudret's ammonical, 377
Bladder, case of rupture of the, 691
Botany, medical, 302
Bowditch's young stethoscopist, or the student's aid to auscultation, 416
Bouchardat's nouveau formulaire magistral, 553
Bostock, Dr., death of, 607
Bone, successful removal of portions of, (probably the pubis,) with a large calculus mass from the bladder, 700
Bronchus, case of lodgment and retention of a foreign body in, for sixty years, 210
Bricheteau on the antagonism of ague and of pulmonary consumption, 248
Brodie's clinical lectures on surgery, 289
Breschet, successor to, 332
Braithwaite's Retrospect, 483
Brocchieri water, remarks upon the hemostatic virtues of, 489
Bread without fermentation, 519
Brain, case of tumour in the, 21
Brain, injuries of the, ill results of operative measures in cases of, 632
Breast, female, the structure and functions of the, by E. W. Tuson, F.R.S. F.L.S., 647
Burns, lunar caustic in, 458

Case of fracture of left parietal bone, from the expulsive action of the uterus, by J. D. Wharrie, M. D., 69
Case of varicose aneurism, successfully treated by pressure, 752
Case of calculus vesicæ, dilatation of the urethra, lithotrity, by H. Crawford, Esq., 191
Cazenave and Schedel's manual of diseases of the skin, 45
Cæsarian section, 66
Catalogues, 177
Catalogues and annual announcements, 110
Catalogues and circulars of medical colleges, 572
Cataract in both eyes, case of, 743
Castor oil seeds, 202
Case of cataract in both eyes, 743
Carbonates, alkaline, on the question whether they exist in the blood, 212
Cattle, destructive disease among, 251
Calomel and common salt, injurious effects of the simultaneous administration of, 325
Carpenter's Elements of Physiology, including physiological anatomy, 353
Cancer of the stomach, 361
Caoutchouc, therapeutical application of, 370
Caustics, means of counteracting the action of, 280
Carson's address to the graduates of the Philadelphia Col. of Pharmacy, 416
Calculi of the prostate gland, 505
Caries of the articular extremities of the bones, excision of the elbow-joint, in a case of, 574
Cataract, 629
Centaurine, 202
Cerebral extravasation, case of, in a new born infant, by H. W. Worthington, M. D., 220
Cephaloma cured in the foot, subsequently developed in various internal organs, 261
Chemistry, animal, with reference to the physiology and pathology of man, by Dr. J. F. Simon, 27
Chemistry, a manual of, by R. D. Hoblyn, A. M.. 240
Chemistry of the seasons, spring, summer, autumn, winter, by Thomas Griffiths, 654
Churchill's theory and practice of midwifery, 34
Child's report on vaccination, 95
Chorea, electrical, 202
Chyle, absorption of, experiments and observations on the, 324
Cholera, Asiatic, 379, 419
" introduction of, into Persia, 458
Chalybeate water, formula for extempore gaseous, 380

Chomel on intestinal obstructions, 512
Chimaphila umbellata, on the use of the, in the treatment of fungus articuli, or white swelling, by G. C. Blakey, M. D., 585
Circumscribed pneumonia of the left lung, 310
Circulars, annual, of medical colleges, 569, 603
Clymer's remarks on pellagra, 18
" on fevers, 407
Climate of Madras, 203
Clavicle, resection of the four external fifths of the, 684
Colchicum, observations on, 734
Corns, bunions, disease of the nails, and general management of the feet, 45
Correspondents and publishers, 243
Connecticut state medical society, annual convention of the, 418
Coley's practical treatise on the diseases of children, 466
Coffee, composition and nutritious property of, 494
Cohesive force of water, Prof. Faraday on the, 495
Contraction of prepuce, 518
Cotton, gun, 720
Cottman's case of general erethism, produced by injury of the membrana tympani, 521
Cowgill's case of perotitis, ending in suppuration of the glands and death of the patient, 705
Coxe's writings of Hippocrates and Galen, 561
Cookery, French, Francatelli on, 566
Composition of taurine, 579
Compound fracture of the cranium—loss of a portion of brain—recovery, 753
Colleges, medical, Philadelphia, 659
Crawford, on ectrotic treatment of small-pox, 731
Creasote, chemical relations of, 96
Crosse's treatise on the formation, constituents, and extraction of the urinary calculus, 86
Craddock's case of extra-uterine pregnancy, fœtus carried 22 years, 286
Croup, on the surgical treatment of, 620
Crawford's case of erysipelas, rheumatism, jaundice and abortion, 678
Cure of stammering, 265

Danger of mistaking a detained testicle for bubonocele, 330
Death of the late James Johnson, 76
" of the late Dr. H. Neill, 79
" of a child, from injury dependent on forcible exposure, 376
Delpech, on the nature and symptoms of varicella, 256
Deformities, nature and treatment of, by R. W. Tamplin, F. R. C., S. E. 289
Diseases of workmen employed in the fabrication of lucifer matches, 266
Diseases of the chest, by W. W. Gerhard, M. D., 299
Diseases of children, a treatise on the, by J. M. Coley, M. D., 466
Diseases of the organs of circulation and respiration, an anatomical description of the, by Dr. C. E. Hasse, 470
Director and cutting gorget, by W. R. Wilde, M. D. 333
Dissection, new mode of preserving bodies for, 373
Diarrhœa of infants, clinical lectures on, 490
Dictionary, a pentaglot, by S. Palmer, M. D., 587
Dislocation of the astragalus, 632
" of the humerus, on a new variety of, 686
Domestic physician, by C. Hering, 706
Dropsical affections successfully treated with sugar, 158
Dropsy, ovarian, 251
Dropsy, ovarian, case of, followed by recovery, 267
Dropsy, ovarian, case of, treated by tapping and iodine injection, by B. A. Allison, M. D., 336
Drunkard, death of, once a physician, 146
Durlacher's treatise on corns, bunions, diseases of the nails, and general management of the feet, 45
Dunglison's general therapeutics and materia medica, 171
Dunglison's dictionary of medical science, 415
Dunglison's human physiology, 568
" new remedies, 656
Durrant's case of tracheitis, condensation of the lower third of the left lung, 309
Dublin Quarterly Journal of medical science, 348
Duration of pregnancy, an important law case, 381
Duclos on fissure of the anus in infants, 510
Duodenum, instance of perforation of the, 628

Ectrotic treatment of small-pox, 717
Editorial, 46, 109, 175, 243, 300, 356, 417, 480, 569, 602, 669, 717
Electrical chorea, 202
Electro-magnetism, a case of hydrocele cured by, 368

Elliott, Com. Jesse D., report of the case of, by E. K. Kane, M. D., 273
Ellis's medical formulary, 554
Elbow-joint, excision of, 574
Enema, tobacco, dangerous effects from the use of, 202
Encysted hydrocele of the epididymus, 208
Enterotomy, case of, 633
Epidemic small pox, 47, 109
Epileptic, marriage of an, and murder by, 136
Ergotine in hemorrhage, by M. Bonjean, 741
Errata, 80
Erysipelas, nitrate of silver in, 702
Erethism, general, produced by injury of the membrana tympani, by J. B. Cottman, M. D., 521
Eve's case of compound fracture of the cranium—loss of a portion of brain—recovery, 753
Expulsion of intestines, wound of abdomen, and cure, 752
Extirpation of the uterus, 331
Exchanges, 357
Excision of the upper end of the femur in morbus coxarius, 642
Eye, a manual of the diseases of, by S. Littell, M. D., 168

Fallopian tube, effects of obliterating the, 193
Face, tetanic, 261
Fever, the African, 69
" five dissertations on, by G. Fordyce, M. D., 414
" puerperal, contagiousness of, 435
Fevers, their diagnosis, pathology and treatment, by M. Clymer, M. D., 407
Femurs, simultaneous dislocation of both, 250
Fenner's report, (extract from) on salicine, in the treatment of intermittent fever, 133
Fenner's letters from the north, 602
Fissure of the anus in infants, 510
Fistulous abscess of the liver, case of, 514
Fleming's inquiry into the physiological and medicinal properties of the aconitum napellus, 542
Flourens, M., 573
Forbes' homœopathy, allopathy, and young physic, 221
Food, vegetable and animal, 575
Fracture, partial, of the neck of the thigh bone, account of a specimen of, 122
Fracture of the sacrum impeding delivery, 263
Fractures in ricketty subjects, remarks on, 264
Francatelli on French cookery, 566
Fracture of the lower end of the fibula, 510
Fracture of the humerus, 442
French government and Bichat, 300

General therapeutics and materia medica, by R. Dunglison, M. D., 171
Gerhard on diseases of the chest, 299
Gelatine, to cover pills or extract of copaiba with, 683
Glass pessary broken in the vagina, 194
Glossitis, a case of acute, 450
Griffin's medical and physiological problem, 160
Graduates of the Philadelphia college of Pharmacy, address to the, by J. Carson, M. D., 416
Great subjects, small books on, 565
Griffiths' chemistry of the four seasons, spring, summer, autumn, winter, 654
Gross's elements of pathological anatomy, 106
Gun-cotton, 720

Hanging, case of, treated successfully by the affusion of cold water, 377
Hairy production on the surface of the tongue, 266
Hartshorne, on the use of phosphate of ammonia in rheumatism, 397
Hasse's anatomical description of the diseases and of the organs of circulation and respiration, 470
Harvey, life of, 747
Hernia, umbilical, treated with adhesive bands, 138
Hernia, strangulated inguinal, 454
" case of strangulated, 642
Hensley's experiments on the temperature of bodies after death, 149
Health of Philadelphia, 175, 243, 481
Heart, gunshot wound of the, 252
" morbid condition of the, 368
Hemostatic virtues of the brocchieri water, remarks upon the, 489
Hemorrhage, ergotine in, 741
Hering's domestic physician, 706
Hippocrates and Galen, 417
Hippocrates and Galen, Coxe's writings of, 561
Hospital practice, cases in, 195
Hospital (Pennsylvania) for the insane, report of the, for the year 1845, 225
Hoblyn's manual of chemistry, 240
Horner's special anatomy and histology, 657
Homœopathy, mesmerism, &c., 419

Homœopathy, allopathy, and young physic, by J. Forbes, M.D., F.R.S. 221
Human teeth, the natural history of, by Jos. Fox, M.D., C.L.S.L., 174
Hughes's clinical introduction to the practice of auscultation, &c., 293
Humerus, fracture of the, without the application of violence, 442
Humerus, on the displacement of the lower fragment in fracture of the surgical neck of the, 684
Humerus, on a new variety of the dislocation of the, 686
Hyperæsthesia, excessive case of, by H. H. Fox, 16
Hydrocele, difficult diagnosis, 208
Hydrocele, encysted, of the epididymus, 209
Hydrocele, a case of, cured by electro-magnetism, 368
Hydatids of the uterus, case of, coincident with pregnancy, 358
Hydatic tumour of the womb, 630
Hymen, two cases of imperforate, 686

Illegitimate children, mortality of, 503
Immobility of the lower jaw, 638
Imperforate hymen, two cases of, 685
Introductory lectures, 110
Intermittent fever, salicine in the treatment of, 133
Intermittent fever, on the use of arsenic in, 137
Insanity from hunger, fear and suffering, 137
Insanity, statistics of, 203
Insanity, on man's power over himself to prevent or control, by Rev. J. Barlow, A. M., 596
Insanity, a case of moral, 624
Insensibility during surgical operations produced by inhalation, 719
Injurious effects of the simultaneous administration of calomel and common salt, 325
Interments, premature, 332
Intestines, small, ulceration of the, 260
Infanticide, contusion and flattening of the head, 375
Infants, clinical lectures on diarrhœa of, 490
Intestinal obstructions, 512
Injuries of the brain, ill results of operative measures in cases of, 632
Instruments, lithotritic, on breaking and bending of, 687
Iodine, adulteration of, 380
Iodic acid, test on the fallacies connected with the, 625
Ireland, surgical society of, 253
Italy, medical institutions of, 445

Jackson, on the ectrotic treatment of small pox by tincture of iodine, 464
Journal of medical science, Dublin quarterly, 347
Journal, new medical, 356
Johnson, James, death of the late, 76

Kane's report of the case of the late Com. Jesse D. Elliott, 273
Kane on kiestein, 485
Kidneys, a curious effect of acid irritation of the, 378

Laënnec's treatise on mediate auscultation, and on diseases of the lungs and heart, 343
Language, loss of, observations suggested by two cases of, 693
Lectures, introductory, 110
Lectures on some of the more important points in surgery, 665
Letter from Paris, 322
Life of Harvey, 747
Livezey, on the treatment of bilious remitting fever, 94
Littell's manual of the diseases of the eye, 168
Liston's elements of surgery, 289
Liston's lectures on the operations of surgery, and on diseases and accidents requiring operations, 290
Ligature of the posterior tibial artery of a wound, 314
Liabilities of the muscle in disease, 457
Lithotritic instruments, breaking and bending of, 687
Longshore's account of an enormous abdominal tumour, 217
Lobelia inflata, on the uses of, by A. Livezey, M. D., 405
Lower jaw, immobility of the, 638
London, medical schools in, 693
Lung, left, case of abscess of the, &c. &c., by A. B. Smith, M. D., 197
Lunatic asylum at Worcester, thirteenth annual report of the managers of the State, (Mass.) 225
Lunatic asylum, third annual report of the managers of the State, (New York) 225
Lunar caustic in burns, 485
Ludlow's manual of examinations upon anatomy and physiology, &c., 658

Masse's pocket atlas of the descriptive anatomy of the human body, 44
Madras, climate of, 203
Materia medica and therapeutics, elements of, 242
Mahommedan physicians, 269

Materia medica, manual of, by Dr. F. Oesterlen, 553
Materia medica and therapeutics, retrospect of, 608
Malformation of the uterus, 259
Matches, lucifer, diseases of workmen employed in the fabrication of, 266
Maid, the electric, 267
Matico, on the use of the, by W. S. W. Ruschenberger, M. D., 401
Magneto-electricity, as a parturient, 484
Mal-practice, suit for, 641
Meigs' two cases of double vagina, 703
Medical profession, address to the, in relation to the objects of the national medical association, by the committe appointed for that purpose, 748
Medical graduates in the University of Dublin, regulations for, 744
Medical matters in Paris, 13
" intelligence, New York, 48
" schools, new, 109, 176
" reform, 147
" " French views of, 204
" and physiological problems of, by W. Griffin, M. D., 160
" convention, national, 244, 300, 358, 388, 480
" convention, Ohio, 572
" school of Buffalo, N.Y., 606
" colleges, statistics of, 607
" students and graduates, 302
" botany, 302
" science, a dictionary of, by R. Dunglison, M. D., 415
" institutions of Italy, 445
" practice, Syrian, 449
" formulary, Ellis's, 553
" colleges, Philadelphia, 659
" society, N. York, and quackery, 660
" institute, private, 664
" schools in London, 696
Medicine, state of, in Spain, 125
Medical classes in Philadelphia, 717
Medical colleges, Scotch, 744
Mesmerism, 504
" homœopathy, &c., 319
Mendenhall, on the olfactory powers of the turkey buzzard, 339
Medico-Chirurgical society of Edinburgh, 435
Menstruation in an infant, 121
Meigs's case of cellulo-vascular and fibrous polypus, 643
Metallic bodies struck by lightning, production of sulphurets on, 681
Midwifery, theory and practice of, by Churchill, 34
Miasmatic fevers, nature and treatment of, by G. L. Upshur, M. D., 81
Miasmatic diseases of the South, report on the use of sulphate of quinine in, by W. H. Van Buren, M. D., 139
Minor surgery, or hints on the every day duties of the surgeon, by H. H. Smith, M. D., 716
Mitchell, on the therapeutical properties of iodine, 459
Mortality, awful, on board H. M. steamer Eclair, 68
Mortality of Lowell, 270
" of illegitimate children, 503
Morbid condition of the heart, case of, 368
Mounier's case of superfœtation, 622
Muscular contraction, phenomena of, 323
Musk-pods, spurious, new method for detecting, 359
Muscle, liability of the, in diseases, 457
Murphy's lectures on natural and difficult parturition, 712
McGirr's case of traumatic tetanus, in which ice was beneficially applied to the skin, 213

National medical convention, 244, 300, 358, 388, 480
National convention, report of a committee on the subject of a, to be held in the city of New York, 270
Names, alterations of, 417
New York Medical Intelligencer, 48
" " " society and quackery, 660
New medical school, 109, 176
" publications, 176
" symptom in pneumonia, 327
" medical journal, 356
" mode of preserving bodies for dissection, 373
" compound of chlorine, iodine and mercury, in scrofula, 503
Neill, Dr. H., death of the late, 79
" on outlines of the nerves, 105
Needles, extraction of, from the flesh, 210
Neuralgia of the uterus, 631
Nostrum certifiers, 357
Nurses and infants, clinical lectures on diseases of, 362

Observations on the employment of compression in aneurism, by O. Bellingham, M. D., 721
Observations on colchicum, 734
Obstetric medicine and surgery, in reference to the process of parturition, by F. H. Ramsbotham, M.D. 86
Obstetrical cases in recent practice, 485
Odours, case of extreme diffusion of, by A. Wilcocks, M. D. 159
Oesterlen's manual of materia medica 553
Ohio medical convention, 572

Olive-tree, on the use of the bark and leaves of the, 742
Onanism, a few cases illustrative of the ill effects of, 451
Operative surgery, new elements of, by A. A. L. M. Velpeau, 354
Opium, poisoning by, 316
Orbit, tumour of the, 339
Ova, expulsion of, entire, 250
Ovarian dropsy, 251, 267

Parietal bone, fracture of the left, from the expulsive action of the uterus, 69
Pathological anatomy, elements of, by S. D. Gross, M. D. 106
Pathological anatomy, 200
Pathology—curious alteration of the cornea in hydrocephalus acutus, 200
Paralysis, rheumatic, of nurses, 364
Paulus Ægineta, the seven books of, by F. Adams, 561
Palmer's pentaglot dictionary, 587
Paton, on the perceptive power of the spinal chord, as manifested by cold-blooded animals, 420
Parotitis, ending in suppuration of the glands and death of the patient, by C. A. Cowgill, M. D., 705
Parturition, lectures on natural and difficult, by E. W. Murphy, M. D. 712
Pessary, glass, broken in the vagina, 194
Pellagra, 202
Pelvic abscess, 250
Pelvic abscess, case of, opened through the rectum, 506
Peritonitis, case of, 455
Perforation of the duodenum, instance of, 628
Pennsylvania Coll., annual announcement of the medical department of, 664
Phlegmasia dolens, remarkable case of, 128
Philadelphia, medical classes in, 717
Philadelphia, health of, 175, 243, 481
Physiologie pathologique ou Recherches cliniques, *par* A. Lebert, 239
Physiology, human, by R. Dunglison, M. D., 568
Phlebitis cured by the constant application of snow, 251
Physicians, Mahommedan, 269
Phenomena of muscular contraction, 323
Phosphate of ammonia, 342
Phosphate of ammonia, on the use of, in rheumatism, by H. Hartshorne, M. D., 397
Physiology, elements of, including physiological anatomy, by W. B. Carpenter, M. D., F. R. S., 353
Physician to the Shah of Persia, 458
Phillips, on scrofula, 472
Pharmacy, Philadelphia college of, 664
Phosphorus, the melting point of, 683
Pneumonia, circumscribed, of the left lung, 310
Pneumonia, new symptom in, 327
Porrigo Decalvans, objections to the vegetable origin of, 257
Posterior tibial artery, ligature of the, for a wound, 314
Potassæ nitras, two ounces of, taken in mistake for epsom salts, 742
Poisoning with the berries of atropa belladona, or deadly night-shade, 745
Poisoning by opium, 316
" by arsenic, 688, 690
" by lead shot left in a bottle, 702
Poisons, antidotes to, abstract of a lecture on, 370
Porter's two cases of popliteal aneurism cured by compression of the trunk of the artery at the cardiac side of the tumour, 523
Polypus, case of cellulo-vascular and fibrous, by J. F. Meigs, M. D., 643
Pregnancy, case of uterine, by W. H. Mason, 25
Pregnancy, case of vaginal, 201
" uterine, case of extra, by J. W. Craddock, M. D., 286
" a new sign of, 380
" duration of, an important law case, 383
Preparation of the valerianate of iron, by M. Ruspini, 741
Principles and practice of obstetric medicine and surgery, in reference to the process of parturition, 34
Problem, medical and physiological, by Wm. Griffin, M. D. 160
Practice, hospital, cases in, 195
Practical observations on various forms of dyspepsia, 303
Premature interments, 332
Prostate gland, calculi of the, 505
Prepuce, contraction of, 518
Prosecution and conviction of an American physician for illegally practising as an apothecary in England, 624
Production of sulphurets on metallic bodies struck by lightning, 681
Purpura hæmorrhagica, turpentine in large doses in the formation of, by J. M. Neligan, M. D., 71
Publications, new, 176
Puerperal fever, contagiousness of, 435

Quinine, sulphate of, not absorbed when applied endermically, 267

Quinine, valerianate of, 249
Quinine, sulphate, on the use of, in typhoid fever, by A. N. Bell, M.D. 275
Quinine, sulphate of, in large doses in typhoid fever, 502
Quinine in acute rheumatism, 510

Ranking's report on the progress of anatomy and physiology, 48, 111, 177
Remarks on pellagra, by M. Clymer, M. D., 18
Remarkable case of phlegmasia dolens, 128
Reform, medical, 147
Regulations for medical graduates in the University of Dublin, 744
Report, twenty-fifth annual, of the, Bloomingdale asylum for the insane, by P. Earle, M. D., 225
Report, of the Pennsylvania hospital for the insane, for 1845, by S. T. Kirkbride, M. D., 225
Report, thirteenth annual, of the trustees of the state (Mass.) lunatic hospital at Worcester, 1845, 225
Report of the committee appointed on the subject of a national convention, to be held in the city of N. York, 270
Report, twenty-ninth annual, on the state of the asylum for the relief of persons deprived of the use of their reason, 355
Rectum, stricture of the, 361
Retrospect, Braithwaite's, 483
" of materia medica and therapeutics, 608
Remedies, new, by Dr. Dunglison, 656
Remedy for toothache, 755
Respiration, case of alleged uterine, 682
Rheumatism, on the use of phosphate of ammonia in, by Dr. H. Hartshorne, 397
Rheumatism, acute, quinine in, 510
Ricord, on the tertiary forms of syphilis, and their treatment, 204
Rima glottidis closed by warty vegetations, 258
Ricketty subjects, remarks on fractures in, 264
Rohrer, on the identity of variola, varioloid, and variola vaccina, and on the prophylactic power of the vaccine disease, 153
Royal college of surgeons, 332
Ruschenberger on phosphate of ammonia, 342
Ruschenberger on the use of the matico, 401
Rupture of the uterus, case of spontaneous, 691
Rupture of the bladder, case of the, 691

Sacrum, fracture of the, impeding delivery, 263
Scarlatina in the Bahamas, 327
Scrofula, its nature, cause, prevalence, and principles of treatment, by B. Phillips, F. R. S., 472
Scrofula, new compound of chlorine, iodine, and mercury in, 503
School of anatomy, Philadelphia, catalogue of the, 606
Scotch medical colleges, 744
Section, cæsarian, 66
Seasons, spring, summer, autumn, winter, chemistry of, by Thomas Griffiths, 654
Simon on animal chemistry, with reference to the physiology and patholy of man, 351
Siebenhaar's terminological dictionary, 587
Skin, manual of the diseases of the, by Cazenave and Schedel, 45
Small-pox, ectrotic treatment of, 717
Small pox, epidemic, 47
" ectrotic treatment of, by tincture of iodine, by S. Jackson, M. D., 464
Small books on great subjects, 565
Smith's minor surgery, or hints on the every day duties of the surgeon, 716
Snow, the constant application of, a cure for phlebitis, 251
Spurious musk-pods, new method for detecting, 359
Spinal chord, on the perceptive powers of the, as manifested by cold-blooded animals, 420
Squibb's case of transposition of the thoracic and abdominal viscera, 462
Squibb's case of diseased vena cava, 583
State of medicine in Spain, 125
Statistics of insanity, 203
Stammering, cure of, 265
" on the immediate cause of, and the means of cure, 325
Stricture of the rectum, 361
Stethoscopist, the young, or the student's aid to auscultation, by H. L. Bowditch, M. D., 416
Strangulated inguinal hernia, 454
" hernia, case of, 642
Statistics of medical schools, 607
Sulphate of quinine in typhoid fever, by A. N. Bell, M. D., 402
Sulphate of quinine, report on the use of, in miasmatic diseases of the south, by Dr. W. H. Van Buren, 139
Sugar, dropsical affection successfully treated with, 138
Surgical society of Ireland, 253

Surgery, clinical lectures on, by Sir Benj. Brodie, 289
Surgery, elements of, by R. Liston, Esq., 289
Surgery, operations of, lectures on the, and on diseases and accidents requiring operations, by R. Liston, Esq., 290
Surgery, lecture on some of the important points in, 665
Surgical society of Ireland, 253
Surgical treatment of croup, 620
Submucous division of the sphincter in affections of the anus, 507
Swallowing of pins, 445
Syphilis, tertiary forms of, and their treatment, M. Ricord on, 204
Sydenham Society's works, 351, 470
Syrian medical practice, 449

Tamplin's lectures on the nature and treatment of deformities, 289
Taurine, composition of, 579
Teeth, a popular treatise on the, by Robt. Arthur, 108, 416
Temperature of bodies after death, experiments on the, by Benj. Hinsley, Jr., M. D., 149
Temperature, average medium, thro'-out the year for ten years, 204
Tetanus, traumatic, case of, in which ice was beneficially applied to the skin, by J. E. McGirr, M. D., 213
Tetanus, the periods at which it occurs, 330
Tetanic face, 261
Terminological dictionary, by F. J. Siebenhaar, 587
Therapeutical properties of iodine, by J. K. Mitchell, M. D, 459
Thoracic and abdominal viscera, case of transposition of the, by E. R. Squibb, M. D., 462
Tobacco enema, dangerous effects from the use of, 201
Tongue, hairy productions on the surface of the, 246
Toothache, remedy for, 755
Transylvania university, 175
Tracheitis ; condensation of the lower third of the left lung, 309
Trial for the attempt to procure abortion, 443
Triplets, report of a case of, 698
Turpentine in large doses in the treatment of purpura hæmorrhagica, 71
Tumour in the brain, case of, by E. G. Smith, M. D., 21
Tumour, enormous abdominal, account of an, 217
Tubercular disease in children, 363
Turkey buzzard, on the olfactory powers of the, by N. Mendenhall, M.D. 339
Tumour of the orbit, 337
Tuson, on the structure and functions of the female breast, 647
Two ounces of potassæ-nitras taken in mistake for epsom salts, 742
Typhoid fever, on the use of sulphate of quinine in, by A. N. Bell, M. D., 275, 402
Typhoid fever, sulphate of quinine in large doses in, 502

Ulceration of the small intestines, 360
Umbilical hernia treated with adhesive bands, 138
University of Edinburgh, 520
" of New York, annual announcement of the medical department of, 662
Universal and critical dictionary of the English language, by J. E. Worcester, 587
Upshur, on the nature and treatment of miasmatic fevers, 81
Urinary calculus, a treatise on the formation, constituents and extraction of the, 96
Urinary bladder, case of abscess connected with, 123
Uterine pregnancy, case of, 25
Uterine pregnancy, case of, extra, in which the fœtus was carried twenty-two years, by Dr. J. W. Craddock, 286
Uterine respiration, case of alleged, 682
Uterus, mal-formation of the, 259
Uterus, extirpation of, 331
" case of hydatids of the, coincident with pregnancy, 538
" neuralgia of, 631
" case of spontaneous rupture of the, 636

Vaccination, report on, by Henry T. Child, M. D., 95
Vagina, two cases of double, by Prof. Meigs, 703
Valerianate of iron, preparation of the, 741
Variola, varioloid and variola vaccina, on the identity of, and on the prophylactic power of the vaccine disease, by J. S. Rohrer, M. D., 153
Vaginal pregnancy, case of, 201
Varicose aneurism, case of, successfully treated by pressure, 751
Varicella, nature and symptoms of, by M. Delpech, 246
Valerianate of quinine, 249
Vagus nerve, Weber's experiments on the, 324

Vaccination in France, cause of the failure of, 682
Vegetable origin of porrigo-decalvans, objections to the, 257
Vegetable and animal food, 375
Velpeau's new elements of operative surgery, 354
Vena cava, case of diseased, by E. R. Squibb, M. D., 583
Vogel's histologiæ pathologicæ, 239
Voigt, on the employment of phosphate of ammonia, 289

Warty vegetations, rima glottidis, closed by, 278
Warts, artificial, 270
Walkly, on magnetico-electricity, as a parturient, 484
Water, Prof. Faraday on the adhesive force of, 495
Weber's experiments on the vagus nerve, 324
Wilcocks's case of extreme diffusion of odours, 159
Wilde, on the director and cutting gorget, 333
Wistar's system of anatomy for the use of students of medicine, 658
Worthington's case of cerebral extrusion in a new-born infant, followed by spontaneous recovery, 220
Wound of the penis from the bite of a horse, 456
Wound of abdomen, expulsion of intestines, and cure, 752
Worcester's universal and critical dictionary, 587
Womb, hydatid tumour of the, 630

Xyloidine, gun-cotton, 737

